# DNA Damage Repair-Related Genes Signature for Immune Infiltration and Outcome in Cervical Cancer

**DOI:** 10.3389/fgene.2022.733164

**Published:** 2022-03-03

**Authors:** Xinghao Wang, Chen Xu, Hongzan Sun

**Affiliations:** ^1^ Department of Radiology, Shengjing Hospital of China Medical University, Shenyang, China; ^2^ Department of Surgical Oncology, The First Affiliated Hospital of China Medical University, Shenyang, China

**Keywords:** cervical cancer, immune infiltration, DNA damage repair, prognosis biomarker, TCGA

## Abstract

**Background:** The mechanism of DNA damage repair plays an important role in many solid tumors represented by cervical cancer.

**Purpose:** The purpose of this study was to explore the effect of DNA damage repair-related genes on immune function of patients with cervical cancer, and to establish and evaluate a prognosis model based on DNA damage repair-related genes.

**Methods:** In the study, we analyzed the genes related to DNA damage and repair, and obtained two subtypes (F1 and F2). We selected two groups of samples for different selection, and studied which pathways were enriched expression. For different subtypes, the immune score was explored to explain immune infiltration. We got the key genes through screening, and established the prognosis model through the key genes. These 11 key genes were correlated with the expression of common Clusters of Differentiation (CD) genes in order to explore the effects of these genes on immunity.

**Results:** Through the Least absolute shrinkage and selection operator (LASSO) method, we screened 11 genes from 232 candidate genes as the key genes for the prognosis score. Through the Kaplan-Meier method, four genes (HAP1, MCM5, RNASEH2A, CETN2) with significant prognostic significance were screened into the final model, forming a Nomogram with C-index of 0.716 (0.649–1.0).

**Conclusion:** In cervical cancer, DNA damage repair related genes and immune cell infection characteristics have certain association, and DNA damage repair related genes and immune cell infection characteristics can effectively predict the prognosis.

## Introduction

Cervical cancer is a common gynecologic tumor, which poses a great threat to women’s reproductive health and life safety. The incidence rate of cervical cancer is the fourth malignancy in the world (Sung et al., 2021). It has been established that long time infection of type 16 and type 18 human papillomavirus (HPV) is an important risk factor for cervical cancer (Crosbie et al., 2013), and HPV pathogenesis is closely related to DNA damage repair pathway (Spriggs and Laimins, 2017; Mehta and Laimins, 2018; Wallace, 2020). DNA damage repair response is a network of cellular signaling pathways, which can sense, signal and promote the repair of damaged DNA. This damage may be the result of environmental and endogenous activities, including ionizing radiation, ultraviolet radiation and DNA replication errors (Ciccia and Elledge, 2010). Mutations in DNA repair genes usually result in genomic instability, increased risk of congenital diseases and cancer (Eyfjord and Bodvarsdottir, 2005). The core of DNA damage response is ataxia telangiectasia mutation (ATM) and ATM and Rad3 related (ATR) signaling pathways. Therefore, exploring DNA damage repair is of great value for cervical cancer.

Cervical cancer is mainly treated by surgical resection, chemotherapy, radiotherapy or comprehensive treatment. Early cervical cancer patients after standard treatment, the recurrence rate is low, the survival time is longer, but for patients with advanced cervical cancer, the survival time after treatment is often unsatisfactory (Green et al., 2001; Tewari and Monk, 2014). Genetic analysis (2017) could provide prognostic information to guide new biomarkers to prevent metastasis and recurrence of cervical cancer. At the same time, immune infiltration is correlated with the immune response (Shamseddine et al., 2021) and survival (Yang et al., 2019) of cervical cancer patients. In tumor immunotherapy, immune infiltration plays an important role in tumor control and therapeutic response. Understanding the infiltration of immune cells in tumor is a very important index to guide clinical treatment (Fridman et al., 2012; Finotello and Trajanoski, 2018). It will help to better understand the complex anti-tumor response and guide the effective immunotherapy of cervical cancer.

In our study, we estimated the effect of DNA damage repair-related genes on immune function of patients with cervical cancer, and obtained a prognosis model based on DNA damage repair-related genes. It can effectively stratify the prognosis of patients with cervical cancer, guide clinical diagnosis and treatment, and potentially explore the role of immune infiltration and its mechanism.

## Materials and Methods

### Data Collection and Processing

Raw counts of RNA-sequencing data and corresponding clinical information of cervical cancer were obtained from The Cancer Genome Atlas (TCGA) database (https://portal.gdc.cancer.gov/). Transcriptome data and clinical information, which was including age, grade and TNM stage of 306 cervical cancer patients were also collected at the same time. In the official website of TCGA and previously published articles (Burk et al., 2017), the cohort was described in detail, including primary frozen tumor tissue and blood from cervical cancer women who had not received prior chemotherapy or radiotherapy. The data need not be approved by the ethics committee.

### Establishment of Molecular Subtypes

Using R software package ConsensusClusterPlus (v1.54.0) (Qiu et al., 2021) for consistency analysis, the maximum number of clusters was 6, 100 times repeated, 80% of the total sample. The clustering map was analyzed by R software package pheatmap (v1.0.12), and the genes with variance above 0.1 were retained in the gene expression maps. According to the heat map, we formed two subgroups: F1 and F2.

The expression of mRNA was studied by using the limma package of R software (version: 3.40.2). The adjusted *p* value was analyzed in TCGA or GTEX to correct the false positive results. To further confirm the underlying function of potential targets, the data were analyzed by functional enrichment. Kyoto Encyclopedia of Genes and Genomes (KEGG) Enrichment Analysis is useful for analyzing gene functions and related advanced genome function information. In order to better explore the carcinogenic effect of target genes, the clusterprofiler package in R is used to analyze the go function of potential mRNA and enrich Kyoto Encyclopedia of Genes and Genomes (KEGG) pathway.

### Establishment and Evaluation of Prognostic Model

Least absolute shrinkage and selection operator (LASSO) regression algorithm (Yi et al., 2020) was used for feature selection, and 10 times cross validation was used. R software package glmnet (Qiu et al., 2021) was used for the above analysis. Log-rank was used to test KM survival analysis to compare survival differences between the above two groups or groups. TimeROC analysis was performed to compare the predictive accuracy and risk score of gene damage repair genes. For Kaplan–Meier curves, *p*-values and hazard ratio (HR) with 95% confidence interval (CI) were generated by log-rank tests and univariate Cox proportional hazards regression. We screened key genes through the above methods. *p* < 0.05 was considered as statistically significant.

The key genes were used to explore the role of the gene in prognosis by single factor analysis. Then univariate and multivariable Cox regression analysis were used to show the *p* value, HR and 95% CI of each variable by using forest map through “forestplot” R package. According to the results of multivariate Cox proportional hazards analysis, nomogram was established by R software package “RMS” to predict the 1,3,5-year total recurrence rate. The nomogram provides a graphical representation of these factors, which can be used to calculate the prognostic risk of a single patient by means of points associated with each risk factor.

### Immune Evaluation

We evaluated the immune function (Iglesia et al., 2016) of different molecular subtypes. In order to evaluate the immune score reliably, we used immune econv, which is an R software package integrating six latest algorithms, including timer, xcell, MCP counter, cibersort, epic and quantiseq. These algorithms have been systematically benchmarked, and each algorithm has its own unique performance and advantages.

We studied the correlation between the key genes screened by lasso and the common CD molecular genes, in order to explore their effects on immunity. The two-gene correlation map is realized by the R software package ggstatsplot, and the multi-gene correlation map is displayed by the R software package pheatmap. Spearman’s correlation analysis was used to describe the correlation between quantitative variables without a normal distribution. A *p*-value of less than 0.05 was considered statistically significant.

## Results

### Molecular Subgroup Based on DNA Damage Repair Genes

**FIGURE 1:**
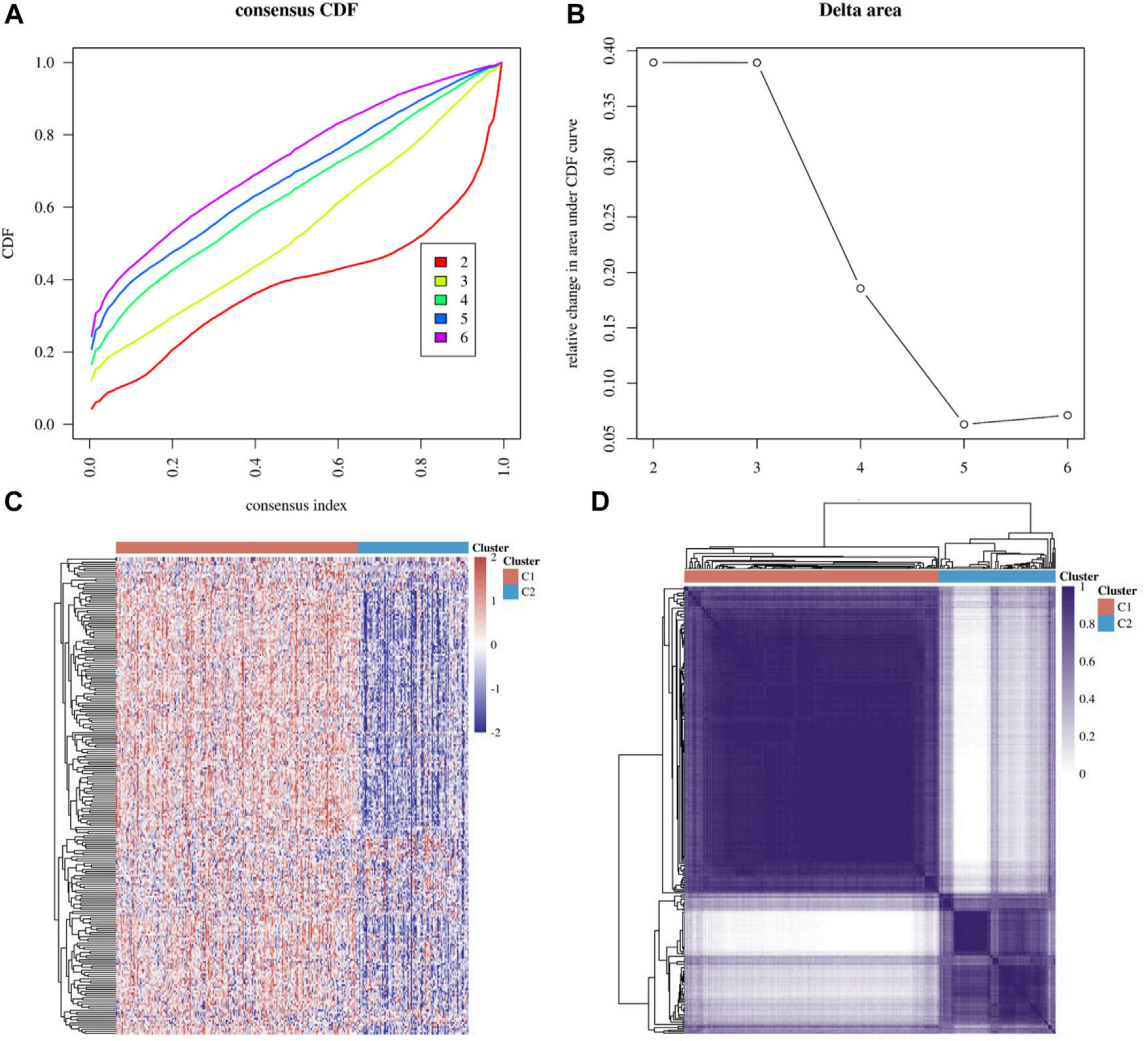
Molecular subgroup based on DNA damage repair genes. **(A,B)** Delta area curve of consensus clustering, indicating the relative change in area under the cumulative distribution function (CDF) curve for each category number k compared with *k*−1. **(C)** Heatmap of DNA damage repair-related gene expression in different subgroups, red represents high expression and blue represents low expression **(D)** Heatmap depicting consensus clustering solution (*k* = 2) for DNA damage repair genes.

**FIGURE 2:**
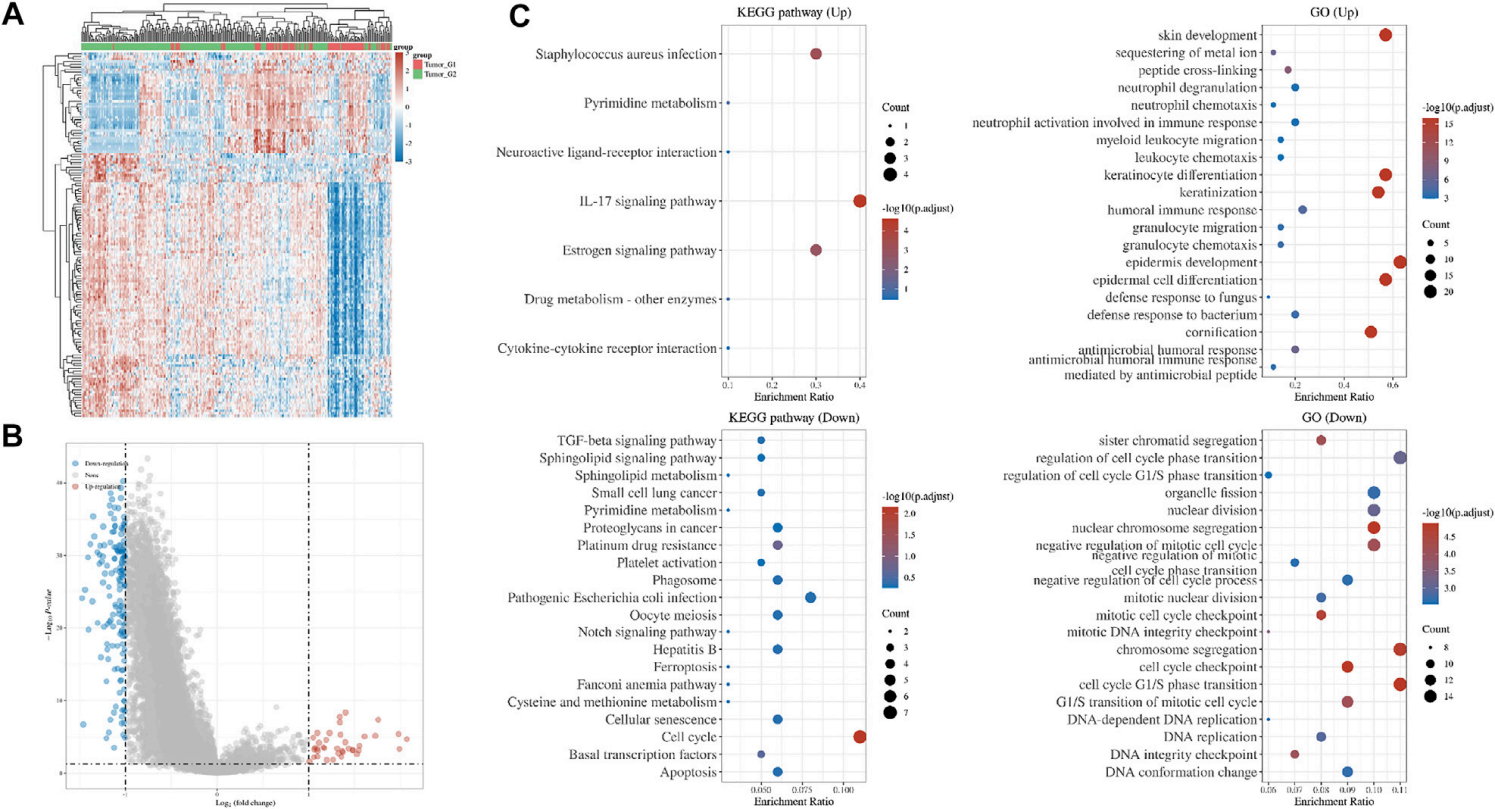
Differences in gene expression between two subgroups. **(A)** Hierarchical clustering analysis of mRNAs, which were differentially expressed between two subgroups **(B)** Volcano plot of two subgroups **(C)** GO and KEGG analysis of two subgroups.

Based on DNA damage repair-related gene expression levels, we identified distinct subgroups of 306 cervical cancer samples. We found that *k* = 2 achieved adequate selection (Figures 1A,B). All patients were successfully categorized into two subgroups in terms of the most stable k value (*k* = 2). By proportion of ambiguous clustering (PAC), we formed two subgroups: F1 and F2 (Figures 1C,D). We found that there was a significant difference in TNM stage between the two subgroups. (Supplementary Table S1). Differentially expressed genes (DEGs), Gene Ontology (GO) annotation and KEGG pathway enrichment analysis between the two subgroups were shown in Figure 2.

### Key Genes

**FIGURE 3:**
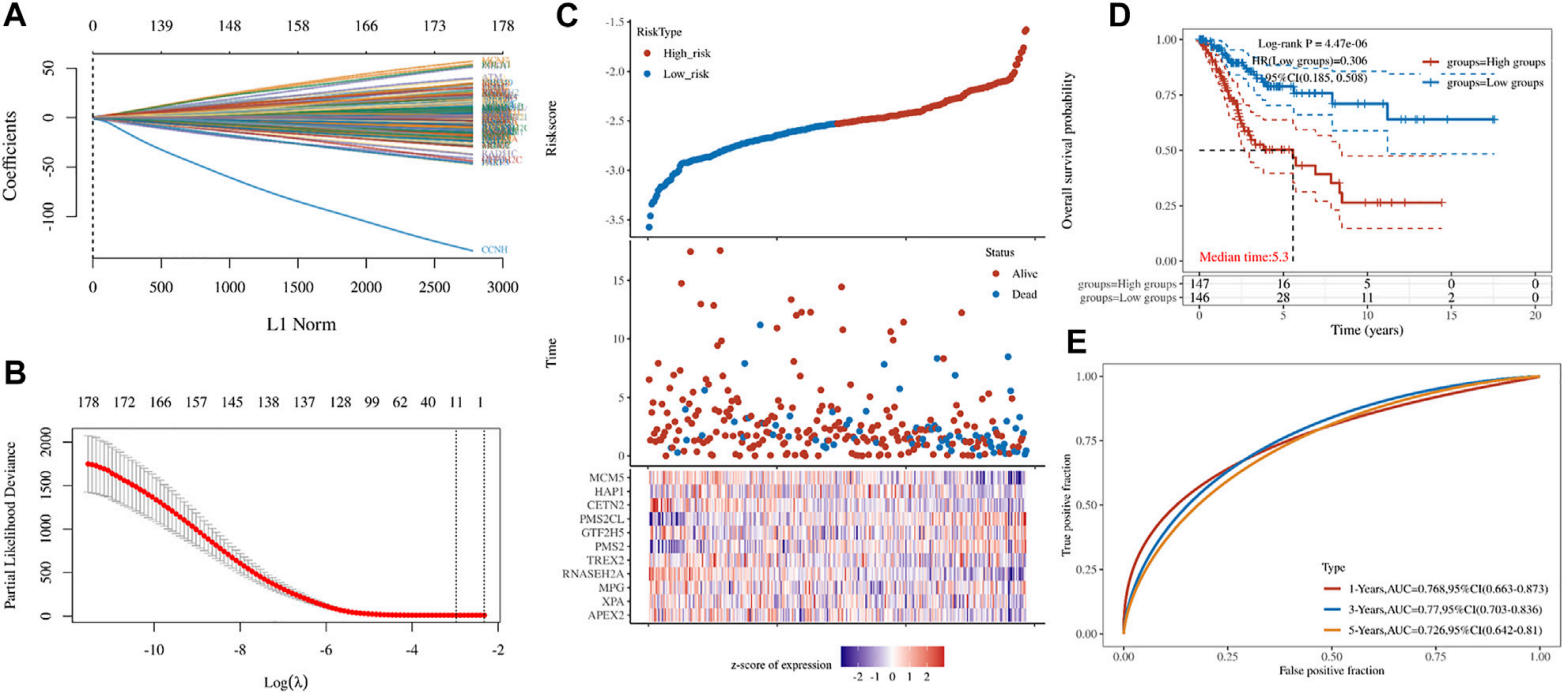
Key genes. **(A,B)** LASSO Cox regression model **(C)** Cervical cancer patients grouped according to Riskscore **(D)** The K-M curve for the different groups of Riskscore **(E)**The ROC of model of Riskscore in 1,2,3-year.

LASSO constructs a penalty function by constructing a penalty function, so that it compresses some coefficients and sets some coefficients to zero. Therefore, LASSO preserves the advantage of subset shrinkage. It can realize variable selection at the same time of parameter estimation, and better solve the multicollinearity problem in regression analysis. In order to screen out the key genes, we constructed a minimum lambda value (0.0511) model, including APEX2, XPA, MPG, RNASEH2A, TREX2, PMS2, GTF2H5, PMS2CL, CETN2, HAP1, and MCM5 (Figures 3A,B). Through key genes, we established a risk model: Riskscore = (−0.0526)*APEX2 + (−0.0331)*XPA + (0.0158)*MPG + (−0.1839)*RNASEH2A + (−0.0266)*TREX2 + (0.0348)*PMS2 + (0.0745)*GTF2H5 + (0.3866)*PMS2CL + (−0.206)*CETN2 + (−0.0203)*HAP1 + (−0.1175)*MCM5 (Figure 3C). According to the different groups of key genes, we found that it has a significant impact on the prognosis of patients (Figures 3D,E).

### Immune Evaluation

**FIGURE 4:**
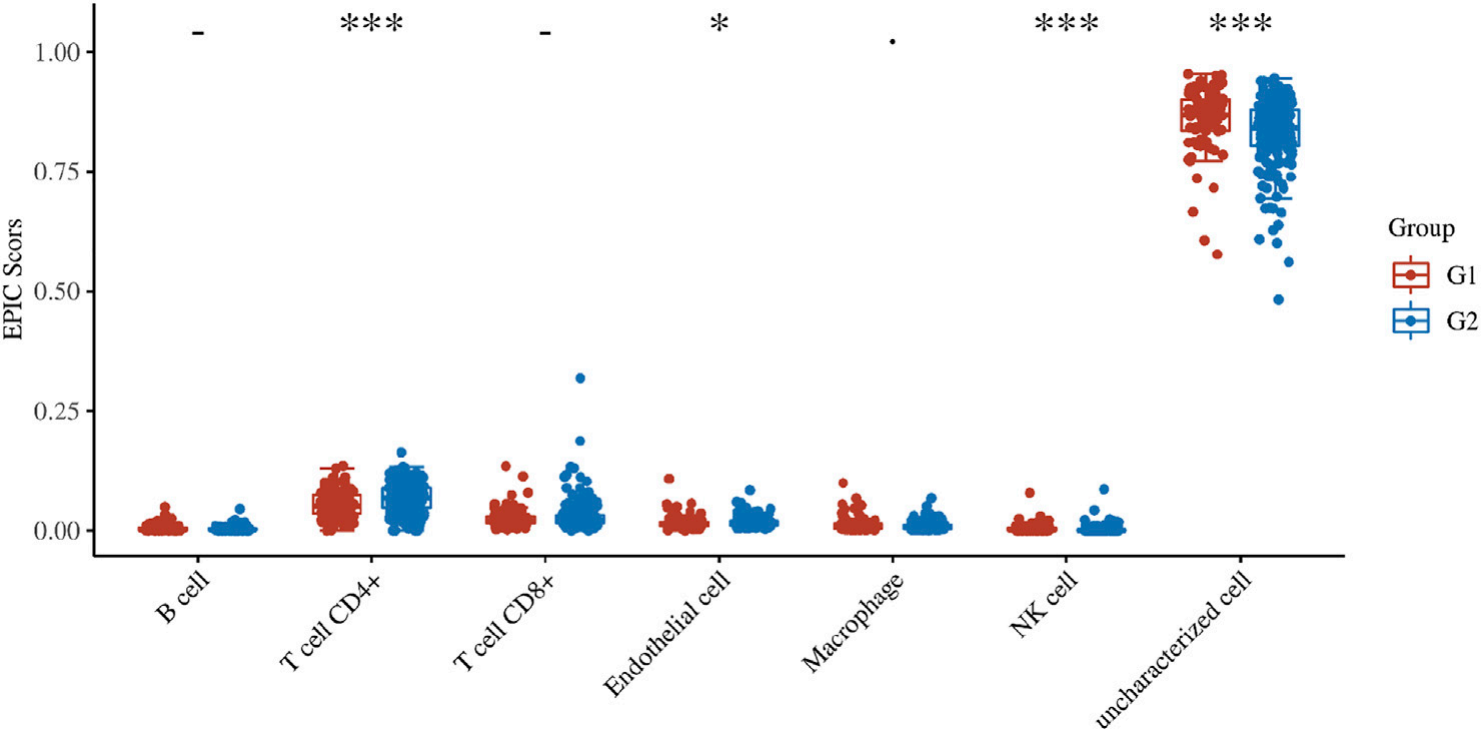
Immune score. The score distribution of DNA damage repair related genes in two subgroups (F1 and F2), the horizontal axis represents different sample groups, the longitudinal axis represents the gene expression distribution, the different colors represent different groups, and the upper left corner represents the significant *p* value test. Asterisks represent levels of significance (*p < 0.05, **p < 0.01, ***p < 0.001).

**FIGURE 5:**
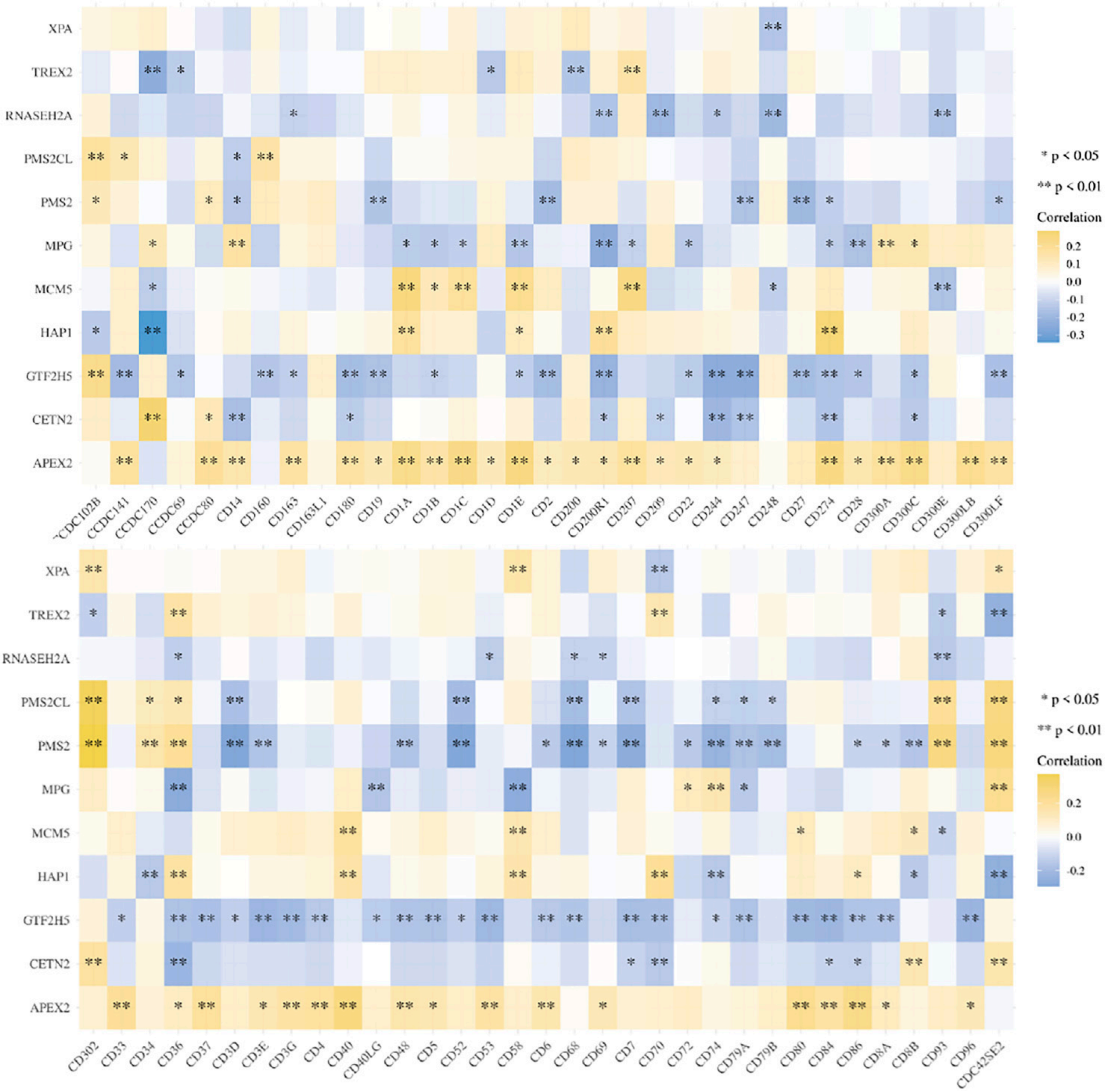
Heatmap of key genes correlation with the CD family.

The genes related to gene damage repair pathway were divided into different molecular subgroups (F1 and F2) by cluster analysis. In order to explore the difference between F1 and F2 in direct immune infiltration, we carried out an immune score. There were significant differences in the infiltration of immune cells between the two subgroups, including CD4 positive T cells, NK cells and endothelial cells (Figure 4). In key genes, we found that many genes were associated with immune related genes (CD family), especially APEX2 (Figure 5).

### Nomogram

**FIGURE 6:**
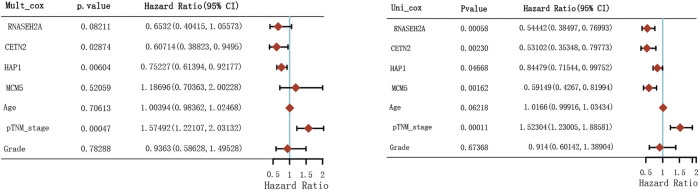
Hazard ratio and *p*-value of constituents involved in univariate and multivariate Cox regression.

**FIGURE 7:**
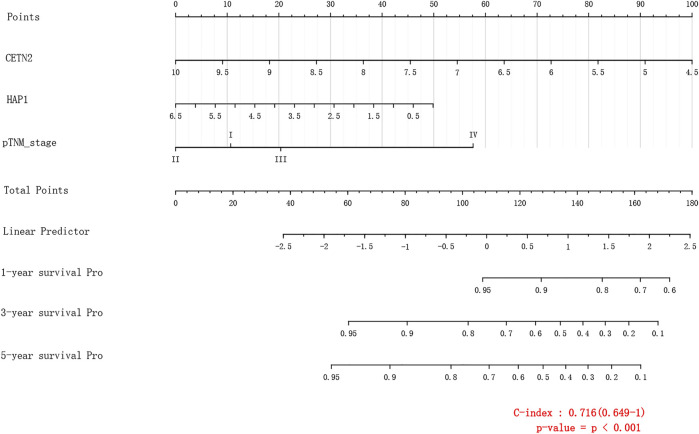
Nomogram.

**FIGURE 8:**
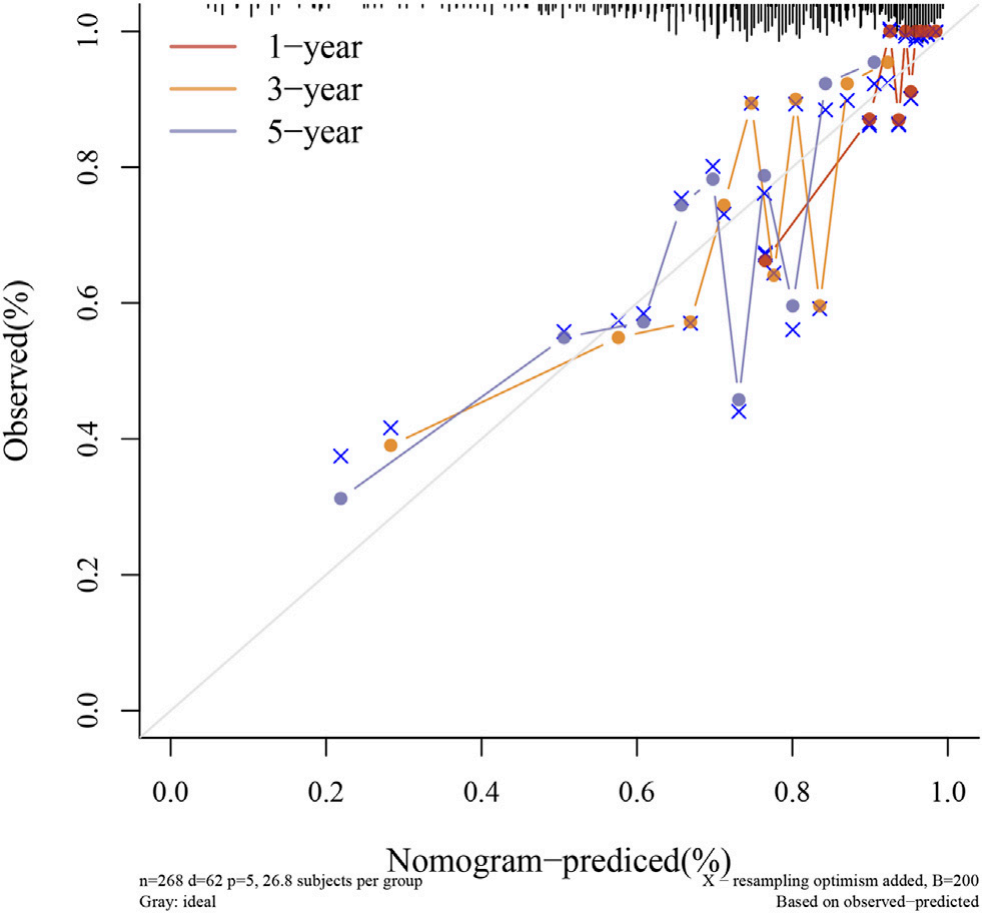
Calibration curve.

Through LASSO, we obtained the key genes which are not collinear and closely related to prognosis. Through the Kaplan-Meier method, four genes (HAP1, MCM5, RNASEH2A, CETN2) with significant prognostic significance were screened out (Supplementary Figure S1). For screening genes, we also described the mutation load of tumor (Supplementary Material S1). Through single factor and multiple factor analysis of these four genes and clinical characteristics, we hoped to find the best prediction model. In univariate analysis, HAP1, MCM5, RNASEH2A, CETN2 and TNM staging were all statistically significant, but HAP1 and CETN2 were shown to be significant in multivariate analysis (Figure 6). The scale is marked on the line segment corresponding to each variable in nomogram, which represents the value range of the variable, and the length of the line segment reflects the contribution of the factor to the outcome event; The point in the figure is the single score, which represents the corresponding single score of each variable under different values, and the total point represents the total score, which represents the total score of the corresponding single scores of all variables; The 1,2,3-year survival prob in the Figure 7 represents the 1,2,3-year survival probability. Finally, the total score can be obtained by adding the scores of various clinical indicators. The 1,2,3-year survival rate of the patient can be predicted in turn. The C-index of the model reached 0.716 (0.649-1). We calculated calibration curves to evaluate models for 1, 3, and 5 years of different life time (Figure 8).

## Discussion

The mechanism of DNA damage repair in cervical cancer is often thought to be caused by HPV infection (Moody and Laimins, 2009). When HPV infects epithelial cells, a variety of pathways or mechanisms related to DNA damage repair are encouraged to ensure the completion of the virus life cycle. E6 and E7 are the most important proteins in HPV, which can bind and inhibit p53 and pRb respectively (Spriggs and Laimins, 2017; Das et al., 2020). The result is that HPV can activate DNA damage repair mechanism for HPV self-preservation, thus hijacking the unit, which will lead to the secondary inhibition of DNA damage repair. The unit is locked in the G1/S phase to allow HPV replication and prevent the cell cycle regulatory functions that normally occur in the presence of DNA damage. Meanwhile, studies have shown that HPV activates ATM (Moody and Laimins, 2009; Sakakibara et al., 2011) and ATR (Reinson et al., 2013; Hong et al., 2015) pathways and fanconi anemia (Spardy et al., 2007; Hoskins et al., 2012) pathways, which are critical pathways in DNA damage repair network. Because of the unrevealed results of cervical cancer genome map (2017), there has been a strong interest in identifying the target molecular pathways for cervical cancer. New therapeutic methods and clinical trials based on DNA damage repair pathway are gradually carried out. At the same time, NCI clinical trials planning meeting report also discussed relevant aspects with the title of “moving forward in cervical cancer: Enhancing sustainability to DNA repair induction and to DNA damage” (Harkenrider et al., 2020). The analysis process of this study was shown in Graphical Abstract. Gene expression and clinical data required for this study were obtained from TCGA database. In previous studies (Shi et al., 2020; Zhang et al., 2020; Cao et al., 2021; Chen et al., 2021; Peng et al., 2021), many scholars used TGCA gene data to predict the prognosis of patients with cervical cancer. In our study, four DNA damage repair related genes (HAP1, MCM5, RNASEH2A, CETN2) were found to be significantly associated with prognosis and can be incorporated into the nomogram to predict prognosis stratification. This is of positive significance in guiding the prognosis of patients.

The screened key genes also deserve our attention. HAP1 (Huntingtin Associated Protein 1) (Zhao et al., 2021) is a Protein Coding gene. Huntington’s disease (HD) was a neurodegenerative disease characterized by loss of striatal neurons, caused by the expansion of polyglutamine beams in the HD protein Huntington protein. The gene encoding a protein interacting with Huntington protein, two cytoskeletal proteins (dynactin and centromere autoantigen protein 1) and tyrosine kinase substrate regulated by hepatocyte growth factor. Interactions with cytoskeletal proteins and kinase substrates indicate that the protein plays a role in vesicle trafficking or organelle transport. Two distinct roles played in cell defense against oxidative stress. A recognized role was to repair all kinds of injuries induced by spontaneous hydrolysis or reactive oxygen species (ROS) in DNA. This function had been described in detail, and the role of amino acid residues in a single active site had been defined. The second function was to regulate the DNA binding activity of a group of nuclear factors, which was related to the pathway of gene damage (Friedberg et al., 2000; Wu and Zhou, 2009). MCM5 (Li et al., 2021) was a member of the MCM family of chromatin binding proteins, which could interact with at least two other members of the family. The encoding proteins were up-regulated in the cell cycle from G0 to G1/S, and may be involved in cell cycle regulation. During the initiation of replication, the eukaryotic replicon was assembled around the CMG helicase at the starting point of replication. Once assembled, CMG shall remain stable with the replication fork until the two fork converges from the adjacent starting point, or the single fork meets the terminal or template discontinuity of the linear chromosome. Among them, the ubiquitination of CMG was inhibited before replication termination to prevent replication fork collapse. This inhibition was mediated by replication fork DNA. Recent study (Jenkyn-Bedford et al., 2021) could have shown that the leucine rich repeat domains of Dia2 and LRR1 are different in structure, but bind to a common site on CMG, including MCM3 and MCM5 zinc finger domains, and LRR-MCM interaction was crucial for the decomposition of replicas, and it is crucial that it is duplicated to exclude DNA chain closure, which lay a structural foundation for inhibiting CMG ubiquitination before termination. Down regulated expression in three negative breast cancer could affect prognosis and chemotherapy sensitivity (Sheng et al., 2021), which could also be seen in other cancers (Lu et al., 2021). RNASEH2A (RNase H2 subunit A) was a protein encoding gene, and its RNASEH2A related diseases include Aicardi-Goutieres syndrome and Aicardi-Goutieres syndrome, associated with cancer progression and cell cycle (Marsili et al., 2021). In our study, it was related to the overall survival rate of cervical cancer, which was consistent with previous studies (Huang et al., 2021), and this gene plays a similar role in lung cancer (Zhang et al., 2021) and breast cancer (Chen et al., 2020). CETN2 belonged to the calcium binding protein family and is the structural component of centrosome. Studies (Mullee and Morrison, 2016; Semer et al., 2019) had shown that CEN2 was a transcriptional co activator, which could reshape the epigenetic landscape of promoters. In previous cancer research, it had received some attention as a potential biomarker (Anurag et al., 2018; Lu et al., 2020).

Traditionally, individual based models are often used to develop predictive and prognostic biomarkers, which requires understanding each patient’s response to treatment and clinical outcomes. In contrast, our method is “unsupervised”, which relies on gene damage related gene expression profiles to reveal the potential structure of the immune landscape in tumors, and also takes into account the immune characteristics. In the future, biomarkers may be developed in combination with the inherent characteristics of the immune landscape. It is conceivable that the stratified model, which first stratified patients into subgroups, and then applied individual based risk stratification, can be used to predict biologically relevant clinical outcomes. The concept of “subtype specific” biomarkers has been successfully applied to improve the prognosis of many kinds of cancers (Hu et al., 2021; Ahearn et al., 2022; Zhang et al., 2022). Therefore, integrating subtype analysis and individual based models may be a promising method to develop clinically relevant biomarkers.

We also explored different immune infiltrating environments between the molecular subsets determined according to the DNA damage repair-related genes (Figure 4). DNA damage repair mechanism can induce innate immune response, resulting in the production of interferon and the potential attenuation of cell proliferation, which is an important way to stimulate immune regulation (Paludan, 2015; Nakad and Schumacher, 2016; Bednarski and Sleckman, 2019). A previous study (Wang et al., 2019) analyzed the proportion of immune cells in cervical cancer and identified the immune cells associated with prognosis. In addition, the mutation load of tumor is also the key internal factor that affects tumor response to immunotherapy (Gasser et al., 2017; Havel et al., 2019). There are many pathways or gene expression related to DNA damage in the work network, which can affect gene instability and affect immunity (Kretschmer et al., 2015; Galsky et al., 2018; Bednarski and Sleckman, 2019; Tuli et al., 2019). The molecular subtypes (F1 and F2) we explored the DNA damage repair-related genes may have potential applications in the search for immunotherapy or immunological checkpoints.

## Conclusion

Through TCGA, we developed a nomogram with clinical application value based on DNA damage repair-related genes, which can better guide clinical prognosis stratification. At the same time, we explored the relationship between DNA damage repair-related genes and immune infiltration characteristics, and made a preliminary inquiry into the immune infiltration of cervical cancer. Of course, the effect of DNA damage repair-related genes on the prognosis and immunity of cervical cancer needs further experimental study.

## Data Availability

Publicly available datasets were analyzed in this study. This data can be found here: The Cancer Genome Atlas (TCGA) database (https://portal.gdc.cancer.gov/).
