# Supplementary figures and images for: DNA Damage Repair-Related Genes Signature for Immune Infiltration and Outcome in Cervical Cancer
Source: Front Genet. 2022 Mar 3;13:733164. doi: 10.3389/fgene.2022.733164 (PMC8927729; doi:10.3389/fgene.2022.733164)

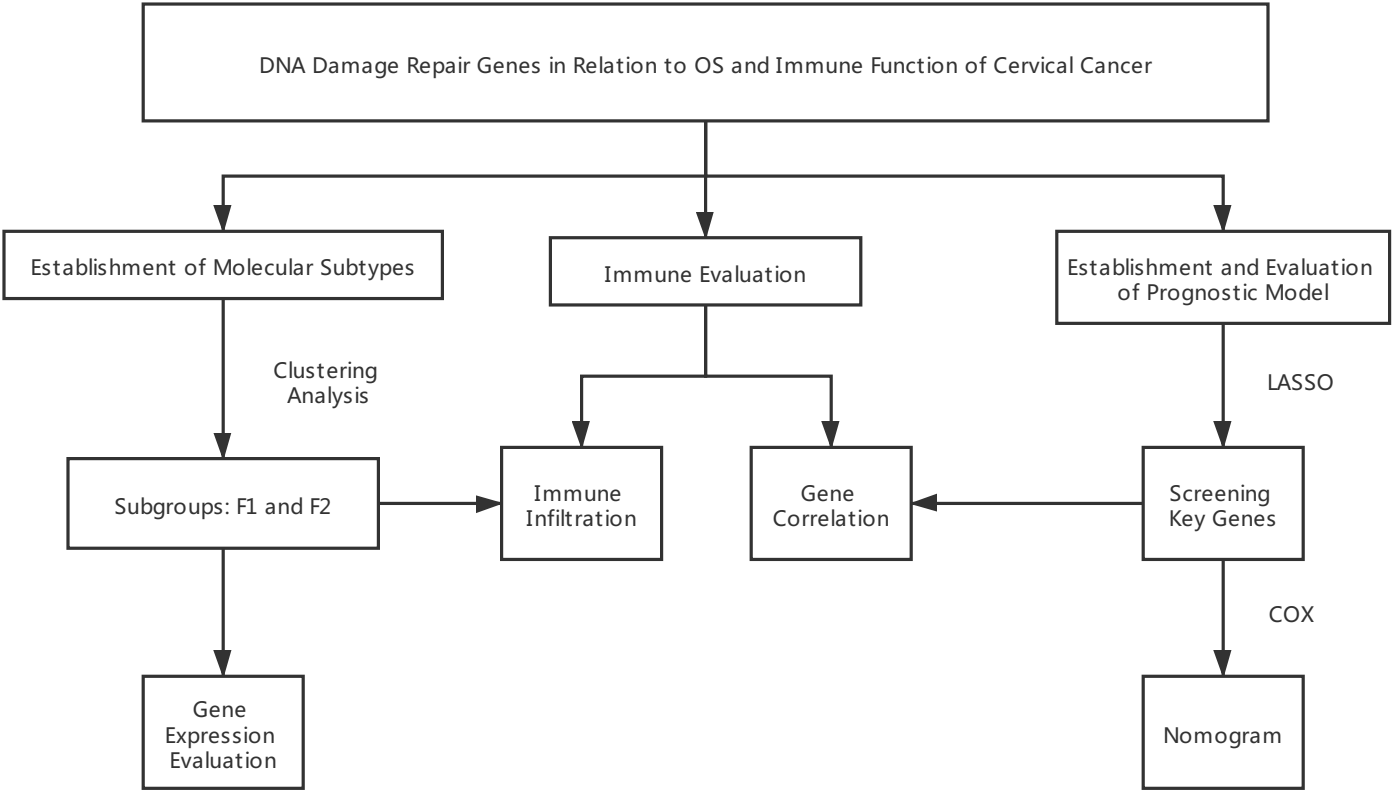

Supplement: Supplementary file 1 [file DataSheet2.PDF]

$\log_e(S) = 12.77$ ,  $p = 0.756$ ,  $\hat{\rho}_{\text{Spearman}} = -0.03$ ,  $\text{CI}_{95\%} [-0.21, 0.15]$ ,  $n_{\text{pairs}} = 127$

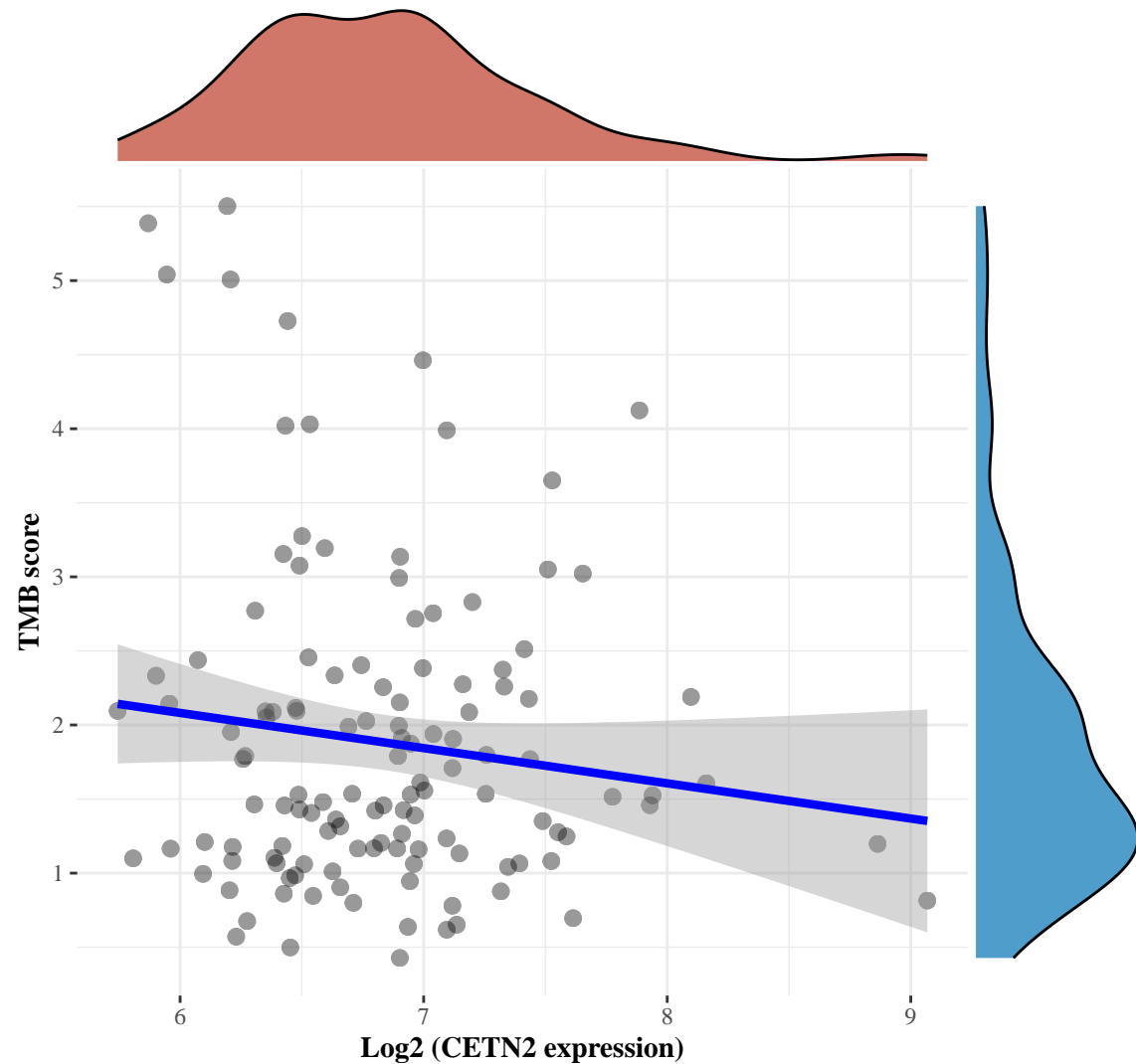

Supplement: Supplementary file 2 [file Presentation1.ZIP › Supplementary material 1/CETN2.pdf]

$\log_e(S) = 12.77$ ,  $p = 0.710$ ,  $\hat{\rho}_{\text{Spearman}} = -0.03$ ,  $\text{CI}_{95\%} [-0.21, 0.15]$ ,  $n_{\text{pairs}} = 127$

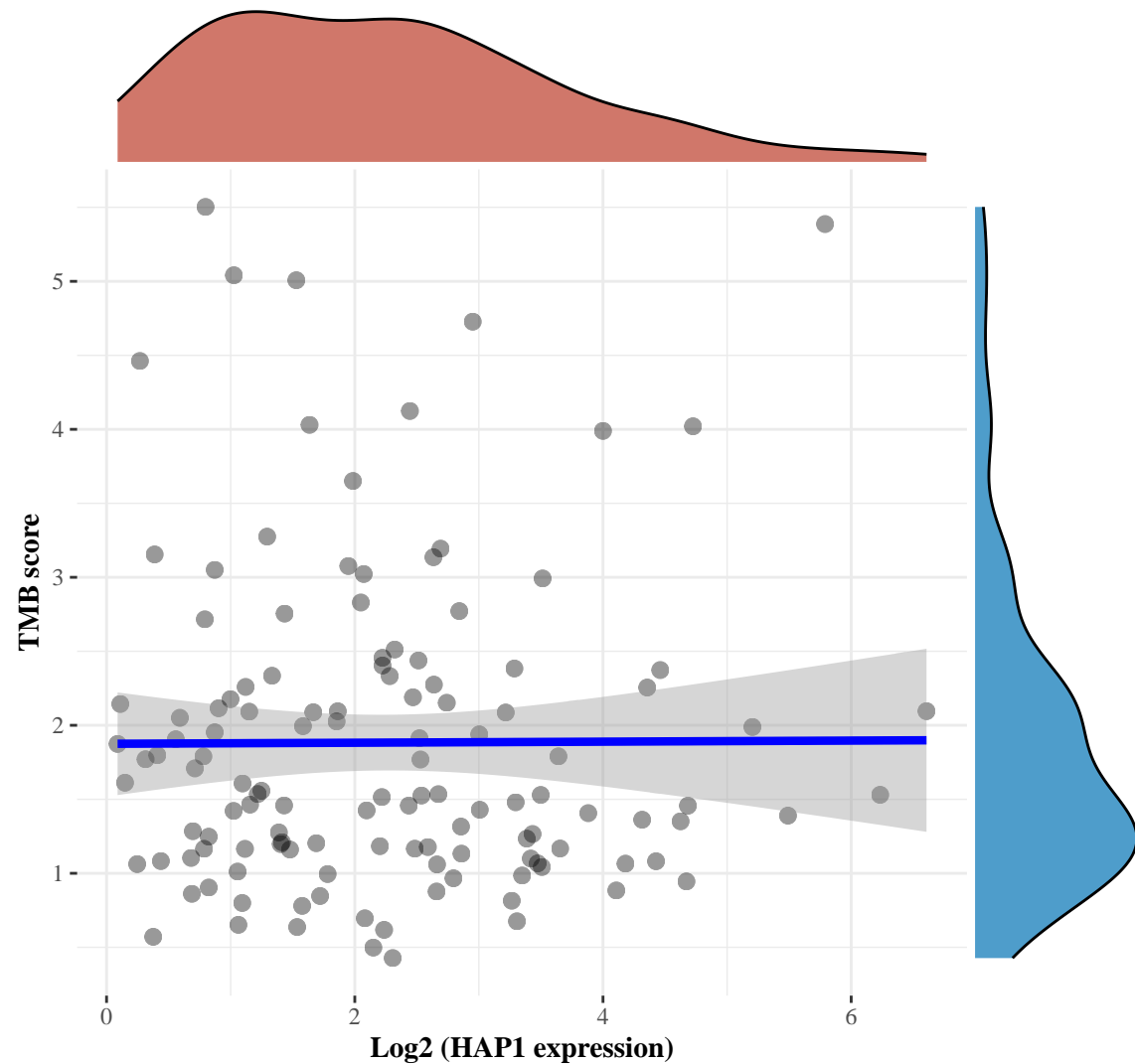

Supplement: Supplementary file 2 [file Presentation1.ZIP › Supplementary material 1/HAP1.pdf]

$\log_e(S) = 12.85$ ,  $p = 0.201$ ,  $\hat{\rho}_{\text{Spearman}} = -0.11$ ,  $\text{CI}_{95\%} [-0.29, 0.07]$ ,  $n_{\text{pairs}} = 127$

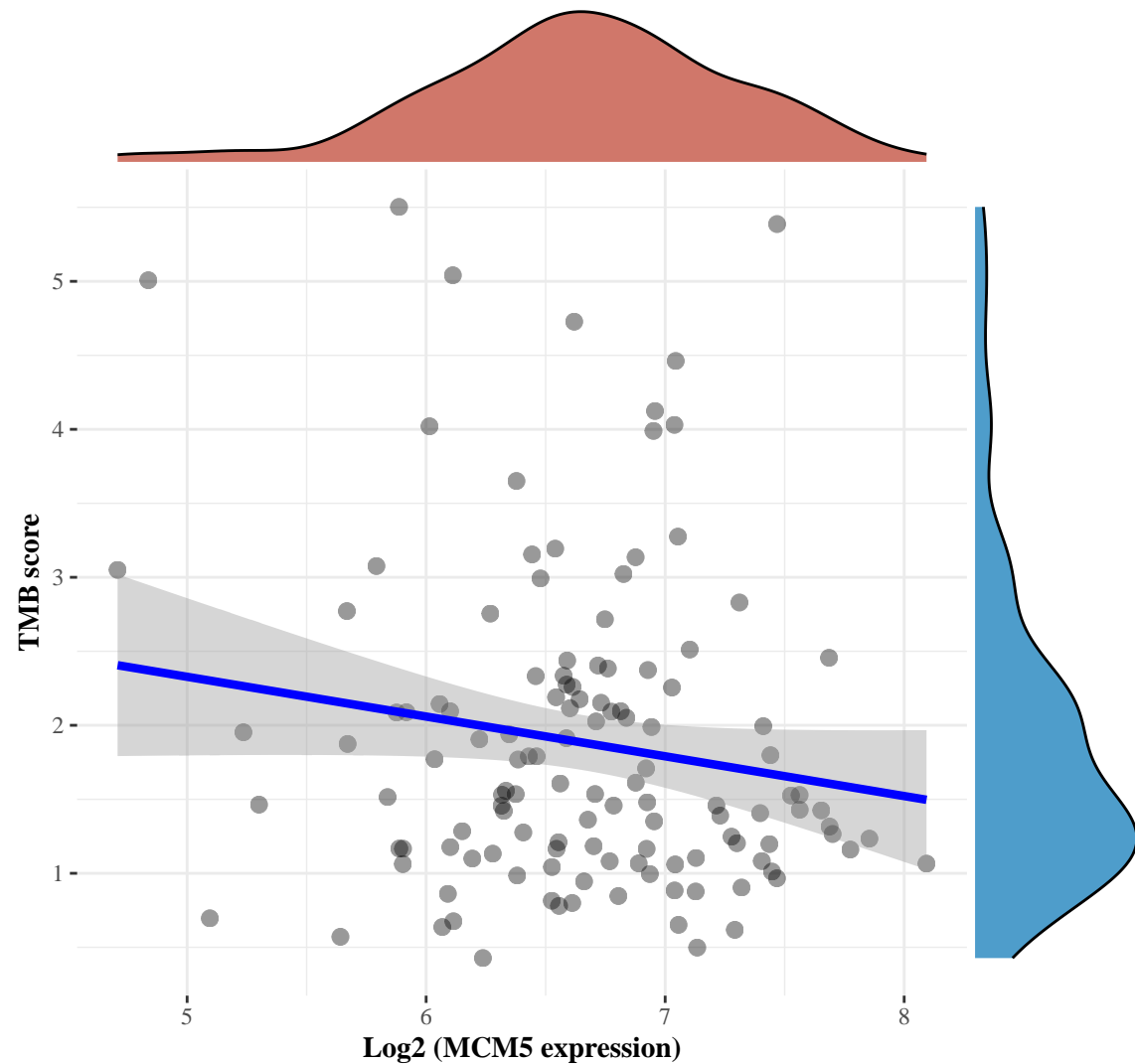

Supplement: Supplementary file 2 [file Presentation1.ZIP › Supplementary material 1/MCM5.pdf]

$\log_e(S) = 12.82$ ,  $p = 0.371$ ,  $\hat{\rho}_{\text{Spearman}} = -0.08$ ,  $\text{CI}_{95\%} [-0.26, 0.10]$ ,  $n_{\text{pairs}} = 127$

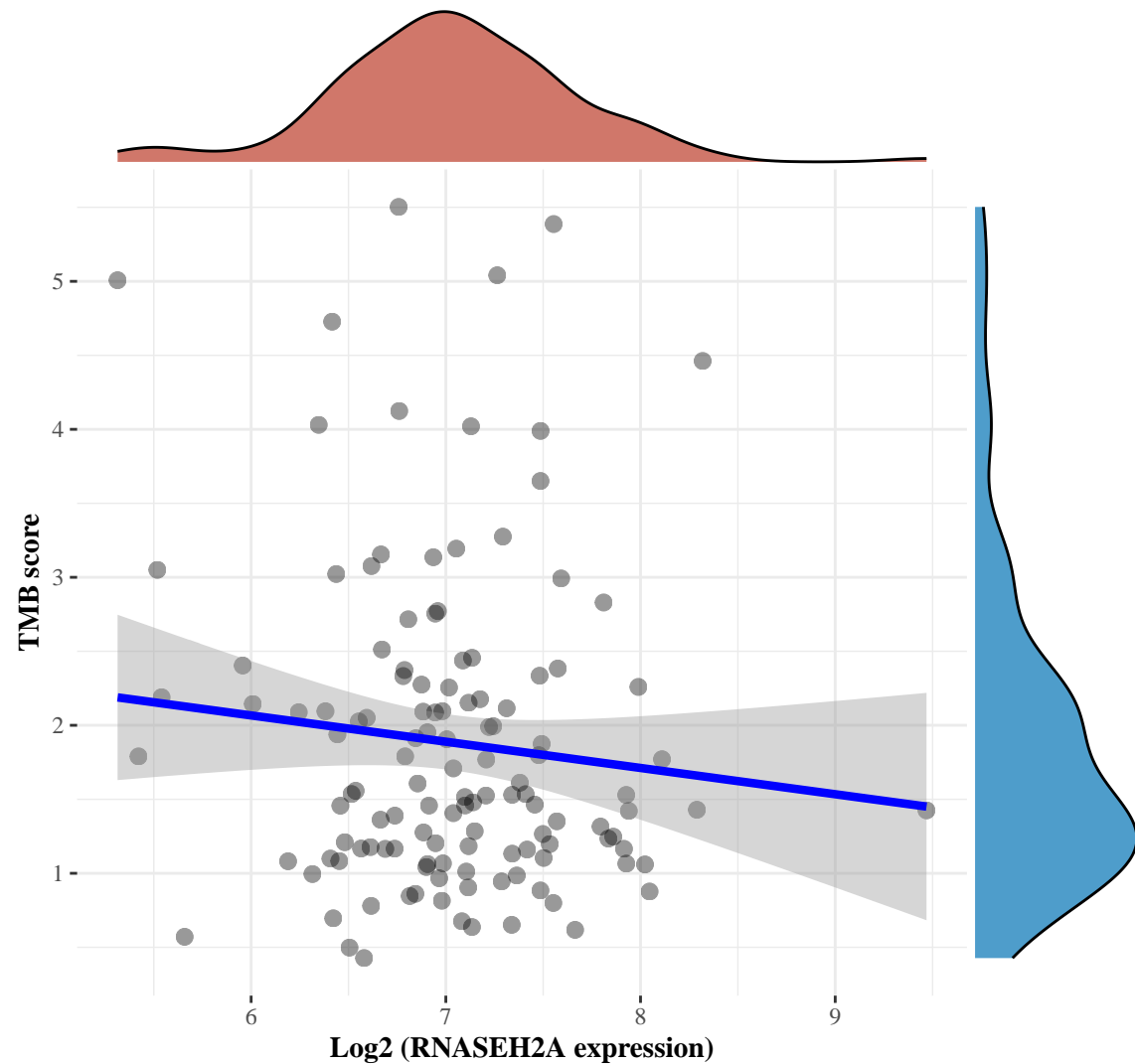

Supplement: Supplementary file 2 [file Presentation1.ZIP › Supplementary material 1/RNASEH2A.pdf]

RNASEH2A

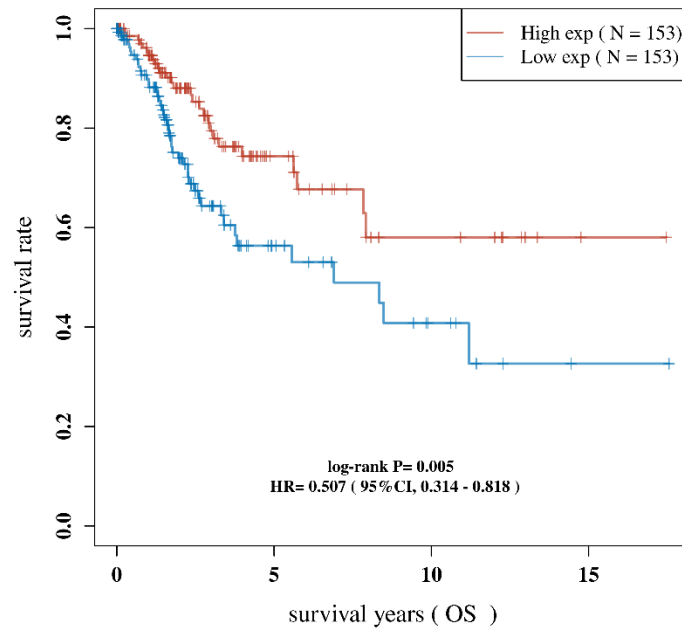

CETN2

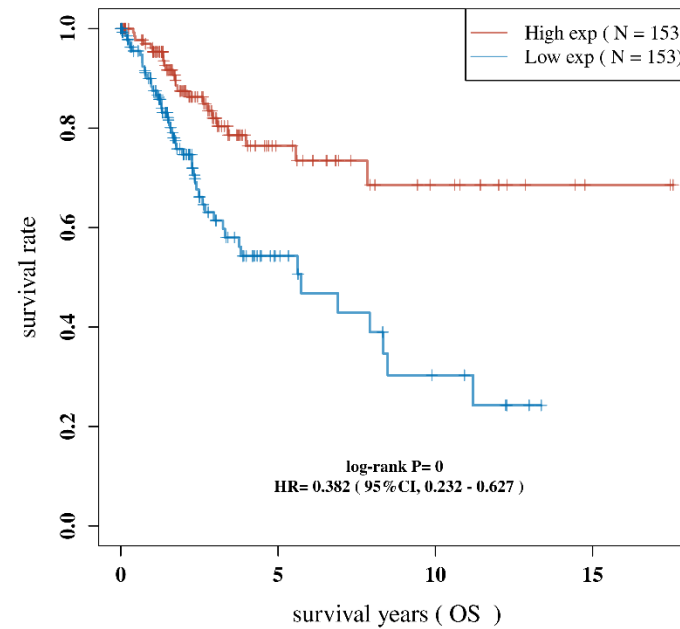

HAPI

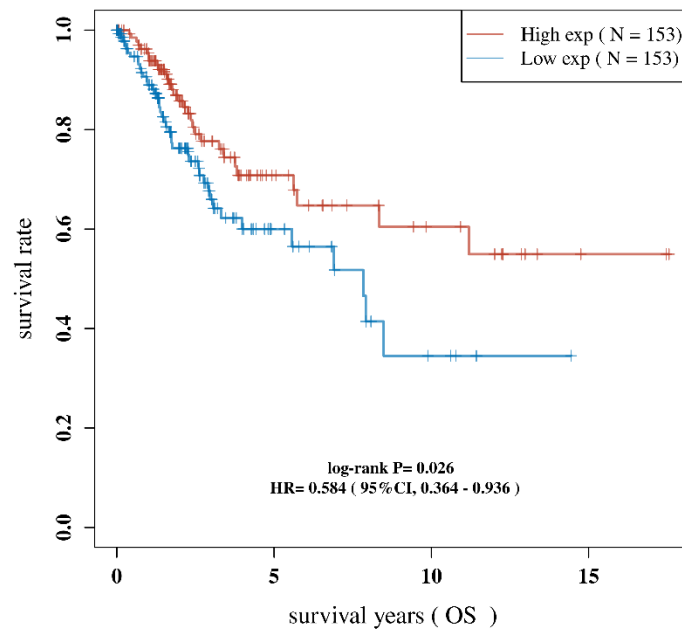

MCM5

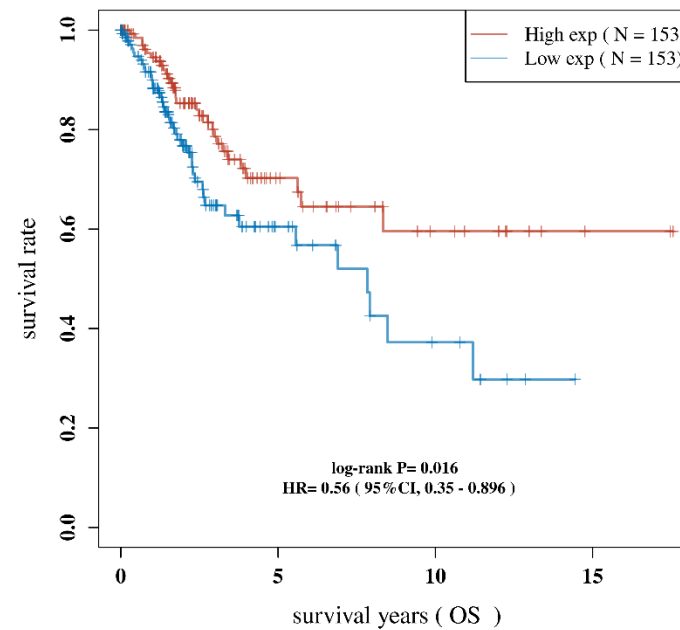

Supplement: Supplementary file 3 [file DataSheet1.PDF]
